# Evolutionary Drivers of Diversification and Distribution of a Southern Temperate Stream Fish Assemblage: Testing the Role of Historical Isolation and Spatial Range Expansion

**DOI:** 10.1371/journal.pone.0070953

**Published:** 2013-08-09

**Authors:** Albert Chakona, Ernst R. Swartz, Gavin Gouws

**Affiliations:** 1 South African Institute for Aquatic Biodiversity, Grahamstown, South Africa; 2 Department of Ichthyology and Fisheries Science, Rhodes University, Grahamstown, South Africa; BiK-F Biodiversity and Climate Research Center, Germany

## Abstract

This study used phylogenetic analyses of mitochondrial cytochrome *b* sequences to investigate genetic diversity within three broadly co-distributed freshwater fish genera (*Galaxias*, *Pseudobarbus* and *Sandelia*) to shed some light on the processes that promoted lineage diversification and shaped geographical distribution patterns. A total of 205 sequences of *Galaxias*, 177 sequences of *Pseudobarbus* and 98 sequences of *Sandelia* from 146 localities across nine river systems in the south-western Cape Floristic Region (South Africa) were used. The data were analysed using phylogenetic and haplotype network methods and divergence times for the clades retrieved were estimated using *BEAST. Nine extremely divergent (3.5–25.3%) lineages were found within *Galaxias*. Similarly, deep phylogeographic divergence was evident within *Pseudobarbus*, with four markedly distinct (3.8–10.0%) phylogroups identified. *Sandelia* had two deeply divergent (5.5–5.9%) lineages, but seven minor lineages with strong geographical congruence were also identified. The Miocene-Pliocene major sea-level transgression and the resultant isolation of populations in upland refugia appear to have driven widespread allopatric divergence within the three genera. Subsequent coalescence of rivers during the Pleistocene major sea-level regression as well as intermittent drainage connections during wet periods are proposed to have facilitated range expansion of lineages that currently occur across isolated river systems. The high degree of genetic differentiation recovered from the present and previous studies suggest that freshwater fish diversity within the south-western CFR may be vastly underestimated, and taxonomic revisions are required.

## Introduction

Understanding the processes that promoted diversification and shaped the distributions of extant taxa is a central question of evolutionary studies [1]–[8]. Studies have cited a plethora of processes, such as global sea-level changes, climatic oscillations, orogenic events, river capture and ecological gradients as the major drivers of diversification and geographical distribution of many freshwater assemblages [5]–[16]. One challenge, particularly in understudied regions, is identifying which of these processes played a major role in shaping patterns of regional diversity. Integrating data from multiple co-distributed taxa provides a more powerful approach for investigating the evolutionary and biogeographical effects of both historical events and the environmental characteristics of a region [15], [16]. The present study uses comparative phylogeographic and biogeographic approaches to examine the evolutionary drivers of diversification and the processes that gave rise to extant geographical distributions of co-distributed stream fishes from the south-western Cape Floristic Region (CFR) of South Africa.

**Figure 1:**
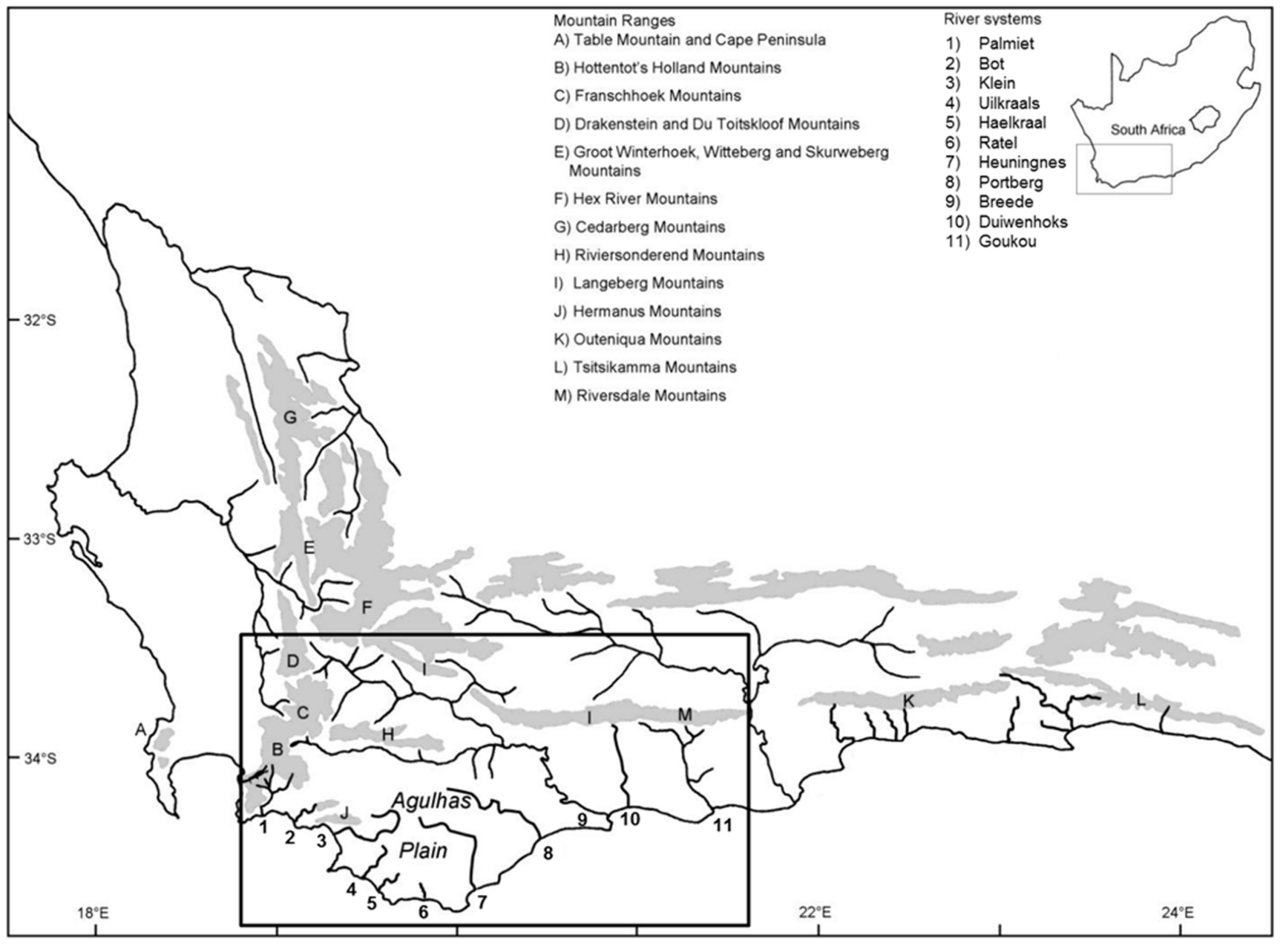
The Cape Floristic Region (CFR) of South Africa showing the Cape Fold Mountains. Location of the south-western CFR and the river systems (1–11) considered in the present study are indicated.

The CFR located at the southern tip of Africa (Fig. 1) is renowned for its high plant diversity and endemism that is unrivalled by other Mediterranean-type ecosystems in the world [17]–[19]. Although the CFR’s ichthyofaunal diversity is much lower, the region is a hotspot of high endemism for freshwater fishes [20]–[22]. Genetic studies are increasingly detecting considerable levels of population structuring in almost all fish species from the CFR investigated thus far [13], [14], [23]–[26]. Many of the newly identified lineages are likely to be described as distinct species, indicating that the region’s taxonomic diversity and endemism has been vastly underestimated [22]. However, knowledge of the mechanisms underpinning diversification and distribution of freshwater taxa in the region is still rudimentary. River capture events and isolation by major mountain barriers have been traditionally proposed as the dominant processes that had major impacts on diversification and distribution patterns of stream fishes in the CFR [27]–[28]. While lineage diversification of freshwater taxa consistent with mountain barriers and river captures can be detected [13], [29], there is an emerging picture that the situation in the CFR has been more complex. For example, a recently identified galaxiid displays phylogeographic patterns indicative of recent range expansion across major mountain ranges and drainage divides [8], while other taxa exhibit a pattern consistent with a model of river confluences during periods of low sea-levels [14].

Southern Africa experienced a complex geological and climatic history marked by tectonism, sea-level fluctuations and extreme wet and dry periods [30]–[37]. The Cape Fold Mountains started to form in the Jurassic (*c.* 140 Ma), but all major present day landform features were probably established by the early Cenozoic (*c*. 65 Ma) [31]. The CFR landscape has been stable since the Post-African uplift events in the Miocene (*c*. 22 Ma) and Pliocene (*c*. 5.3–2.6 Ma) [32], [33]. Southern Africa also experienced repeated climatic oscillations marked by extreme wet and dry conditions since the Oligocene through to the Holocene epoch [34]–[37]. The present dry conditions of the region were probably established by the end of the Pliocene (*c*. 2.6 Ma) [38], [39], but some areas, such as the coastal regions of the southern CFR, are thought to have experienced wetter climatic conditions as recent as the Holocene Altithermal (*c.* 8 000–6 000 years ago) [34].

**Figure 2:**
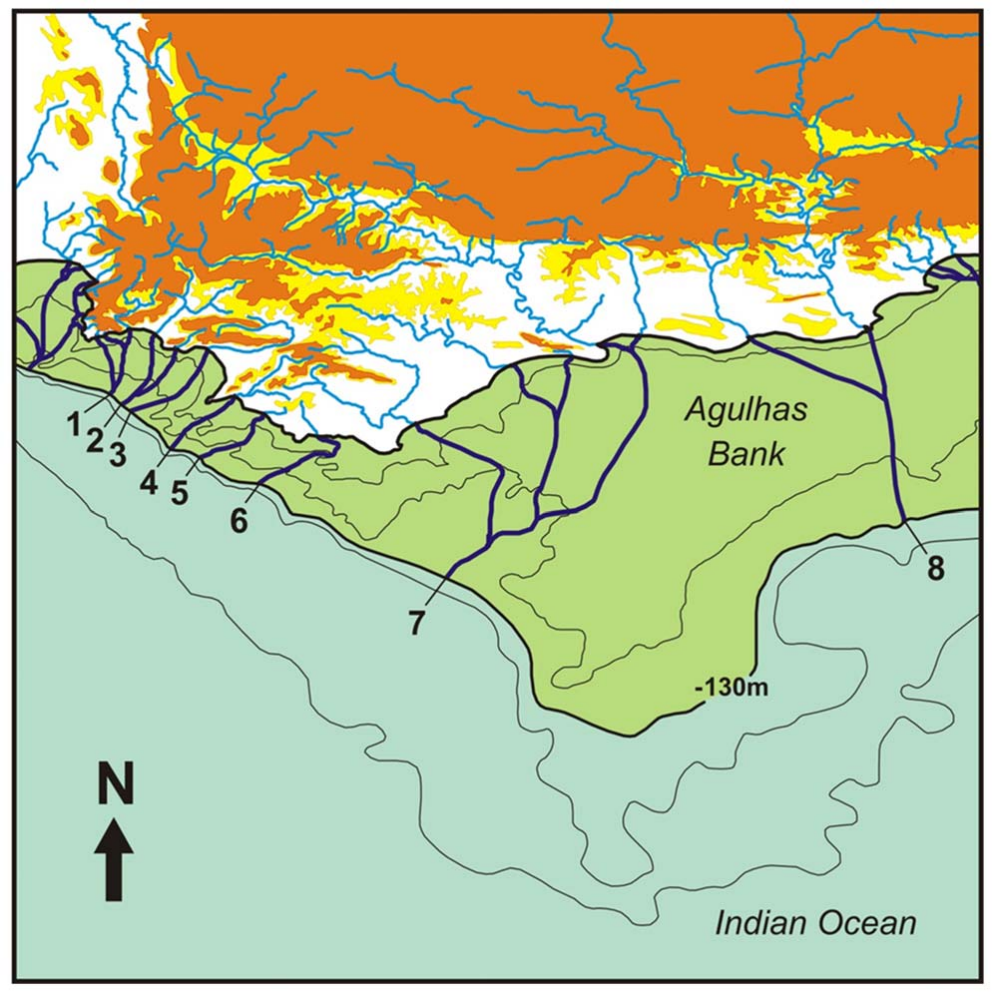
Sea-level changes. Potential fragmentation of river systems during the Miocene-Pliocene sea-level transgression and the proposed palaeoriver systems (1–8) of the Last Glacial Maximum (LGM) in the south-western Cape Floristic Region (CFR). The potential range of the maximum transgression level is indicated by the area in yellow. The white area would therefore have been vulnerable to marine incursion, whereas the area in orange was possibly never affected by the Miocene-Pliocene transgression. The approximate LGM sea-level is represented by the −130 m contour line. The palaeorivers are 1) Palmiet, 2) Bot-Onrus, 3) Klein, 4) Uilkraals, 5) Haelkraal, 6) Ratel, 7) Breede-Heuningnes-Duiwenhoks and 8) Gouritz-Goukou.

Apart from tectonic activity and extreme climatic variability, southern Africa also experienced repeated fluctuations in sea-level throughout the Tertiary as a result of global cycles of glaciation [30], [31], [40]. However, there are uncertainties regarding estimates of the timing and amplitudes of global sea-level changes [40]. This is further compounded by the possibility of differential continental uplift [41], which makes it difficult to assess the true height reached by historical sea-levels. Despite these uncertainties, the documented estimates provide important insights into the relative amplitudes of historical sea-levels [30], [40]. In southern Africa, the last major sea-level transgression occurred from the middle Miocene (*c*. 15 Ma) to the late Pliocene (*c*. 2.6 Ma), with levels being estimated to have reached about 330 m above the current coastline ([30]; Fig 2). The last major regression in southern Africa occurred during the LGM, about 18 ka, when the coastline is estimated to have been about 130 m below the current level ([30], [31]; Fig 2). These processes could have potentially left an imprint in the genetic structure and geographical distribution of stream-dwelling taxa, particularly obligate freshwater fishes.

The south-western portion of the CFR (Fig. 1) is well-suited for evolutionary studies to unravel the impact of palaeogeographic events on the diversification and geographical distribution of stream fishes. This region is clearly demarcated from surrounding areas by the Hottentot’s Holland, Franschhoek, Drakenstein and Du Toitskloof Mountain ranges to the west, the Hex River, Langeberg and Riversdale Mountain ranges to the north, and the Gouritz basin to the east (Fig 1). A unique feature of the south-western CFR is the Agulhas Bank, a shallow continental shelf that would have been periodically exposed and submerged due to repeated fluctuations in sea-level ([30]; Fig 2). Rivers of the south-western CFR have only four currently recognised native primary freshwater fishes: three smaller species [*Galaxias zebratus* Castelnau, 1861, *Pseudobarbus burchelli* Smith, 1841 and *Sandelia capensis* (Cuvier, 1831)] and a large cyprinid barb, *Barbus andrewi* Barnard 1937 [42]. The three smaller species are broadly co-distributed across several river systems, while *B. andrewi* is now largely confined to two man-made impoundments in the Breede River catchment. Molecular studies have revealed existence of historically-isolated lineages within *Galaxias*, *Pseudobarbus* and *Sandelia* in the south-western CFR [14], [43], [44].

**Figure 3:**
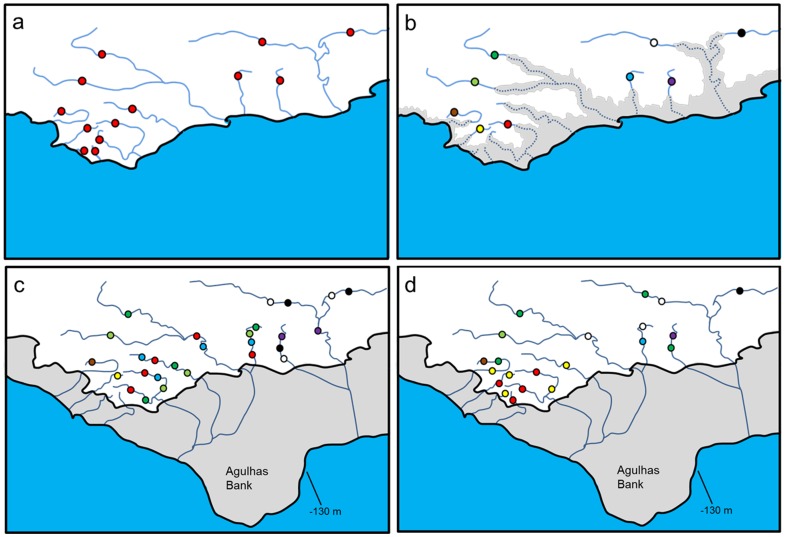
Hypotheses of factors that could have shaped genetic and distribution patterns of fishes in the south-western CFR. Panel (a) assumes that populations could have been historically connected probably during a period of a major sea-level regression. Panel (b) describes the hypothesis that truncation of rivers during the Miocene-Pliocene major sea-level transgression fragmented populations and isolated them into upland areas that were not inundated (Refugia hypothesis), leading to allopatric divergence and the formation of unique lineages (indicated by the different colours). Panel (c) shows the river systems that would have coalesced forming the palaeoriver systems of the Last Glacial Maximum (LGM) (Palaeoriver hypothesis), allowing the exchange of unique lineages between systems sharing a common confluence. Panel (d) illustrates the hypothesis that alternative dispersal routes such as freshwater connections through low drainage divides could have facilitated range expansion of unique lineages (Interdrainage Dispersal hypothesis), leading to unique lineages being distributed across river systems that did not coalesce during the LGM.

The present study extends this previous research by undertaking finer-scale geographic sampling of co-distributed stream fishes belonging to three genera, *Galaxias*, *Pseudobarbus* and *Sandelia*, across 11 river systems in the south-western CFR (Fig 1; Appendix S1), with the aim of assessing the roles of vicariance and population expansion in driving diversification and shaping the present-day distributions of these groups. From a vicariance biogeographic perspective, we hypothesise that the marine incursions during the Miocene-Pliocene (Fig. 2) truncated river systems, fragmenting once widely distributed populations of freshwater fishes (Fig 3a), and isolating them in upland river reaches, potentially driving genetic divergence (Refugia hypothesis: Fig 3b). Under this hypothesis, lineage splitting in *Galaxias*, *Pseudobarbus* and *Sandelia* would be expected to be chronologically associated with the period of the highest sea-level transgression (late Miocene to late Pliocene epochs), and the distribution of unique lineages is expected to show affinities with rivers that were not completely inundated.

From the post-speciation population expansion perspective, we hypothesise that confluence of adjacent rivers during periods of lower sea-levels could have facilitated dispersal of genetically-differentiated lineages (Palaeoriver hypothesis: Fig 3c). Under this hypothesis, genetically-distinct lineages of the same genus would be expected to occur in sympatry across river systems that coalesced during the LGM (Fig 3c). Assuming that genetically-differentiated lineages could have also used alternative dispersal routes such as low drainage divides during periods of heavy flooding [Inter-basin Dispersal hypothesis; 8], we would expect to find a pattern where distinct lineages have broad distributions across rivers that did not coalesce during the LGM low sea-levels (Fig 3d).

Finally, it was predicted that if *Galaxias*, *Pseudobarbus* and *Sandelia* were similarly affected by shared historical events and environmental factors, they would be expected to exhibit congruent geographical patterns and similar dates of lineage splitting. According to Birmingham & Martin [45], evidence of shared history across multiple co-distributed taxa would probably reflect the role of extrinsic climatic or landscape history in shaping contemporary biogeographic patterns, while different patterns would probably reflect the influence of intrinsic biological or ecological differences.

## Materials and Methods

### Ethics Statement

The research was conducted under permit from CapeNature (permit number: AAA-004-000205-0035) issued only after the approval of methods by a review panel.

### Field Sampling

Specimens of *Galaxias*, *Pseudobarbus* and *Sandelia* were collected from 146 localities across the south-western CFR (Appendix S1) between November 2008 and December 2009 using a combination of electric fishing, seine netting, fyke nets and snorkelling with a handnet. Fish were euthanised with clove oil (0.2 ‰). A small piece of muscle tissue or whole specimen was preserved in 95% ethanol. All samples collected for the present study have been deposited at the South African Institute for Aquatic Biodiversity (SAIAB).

### DNA Extraction, Amplification and Sequencing

DNA was extracted from preserved tissue using the Wizard® Genomic DNA purification kit (Promega, USA) following the manufacturer’s protocol. A partial fragment of the mitochondrial cytochrome *b* gene was amplified. For *Galaxias* (*n* = 205), the amplification was done as outlined in Chakona *et al* [8]. The primers GluF and ThrR [46] were used for *Pseudobarbus* (*n* = 177) and the PCR protocol was 94°C for 2 minutes, and 35 cycles of 94°C for 30 seconds, 54°C for 30 seconds and 72°C for 45 seconds, followed by 72°C for 5 minutes. The primers used for *Sandelia* (*n* = 98) were H16091 and L14841 [47] and the PCR protocol was similar to that of *Pseudobarbus*, except that the denaturing and annealing temperatures were 93°C and 55°C, respectively. Sequencing, alignment and editing of sequences were done as outlined by Chakona *et al* [8]. Sequences were submitted to GenBank (accession numbers: *Galaxias* KC821878–KC821890, KC821898–KC821917, KC821922–KC821925, KC821934–KC821938, KC821941–KC821952, KC821954, KC821955, KF222589–KF222667; *Pseudobarbus* KF222577–KF222588, KF222668–KF222791; *Sandelia* KF222792–KF222890).

### Data Analyses

Shared and unique haplotypes for each genus were identified using the program DnaSp 5.10 [48]. Phylogenetic relationships among unique haplotypes within each taxon were inferred using Maximum Likelihood and Bayesian Inference based on the selected model of sequence evolution. The most appropriate model of sequence evolution for each taxon was selected using the AIC in Modeltest 3.7 [49]. The model of sequence evolution selected for *Galaxias* was GTR+I+Γ [50]. For *Pseudobarbus*, the GTR+I model was selected. For *Sandelia* the TIM+I+Γ [51] was the best model selected. *Pseudobarbus tenuis*, *P. asper* and *P. burgi* were used as outgroups for the *Pseudobarbus* phylogeny. *Galaxias* sp. ‘mollis’ (Swartz, unpublished) and *Sandelia* sp ‘Berg’ both from the Leeu River (Berg River system) were used to root the *Galaxias* and *Sandelia* phylogenies respectively.

The ML analyses were done in PAUP4.0b10, using heuristic tree searches and applying the tree-bisection-reconnection (TBR) branch-swapping algorithm with 10 random addition replicates. Bayesian analyses were performed using the Bayesian Markov Chain Monte Carlo (BMCMC) algorithm implemented in MrBayes 3.1.2 [52]. Each analysis was run across four chains for five million generations and sampled every 100^th^ generation to obtain 50 000 sampled trees. The burn-in value was determined by plotting the average standard deviation of split frequencies, tree length and log-likelihood scores against generation time using the program Tracer 1.5 [53]. The first 5000 trees were discarded as burn-in and the remaining trees were used to calculate Bayesian posterior probabilities. For each data set, two separate BI runs were done to assess whether the chains converged to the same point. Model-corrected genetic distances between unique lineages identified for each taxon were calculated using Paup* [54].

### Phylogeographic Patterns

For each genus, genealogical relationships among all samples were also inferred using the statistical parsimony method implemented in the program tcs 1.21 [55]. To test for evidence of recent population expansion, as predicted by the Palaeoriver and Interdrainage Dispersal hypotheses, Fu’s [56] *F_s_* statistic was calculated for each unique lineage with 15 or more individuals using Arlequin [57]. Statistical significance of Fu’s *F_s_* statistic was tested using 1000 random permutations.

### Divergence Time Estimation

Dates of divergence among lineages within *Galaxias*, *Pseudobarbus* and *Sandelia* were estimated using *Beast 1.7.4 [58] based on the respective best models of molecular evolution estimated using Modeltest 3.7. A UPGMA topology was used as the starting tree. A yule tree prior, piecewise linear and constant root population size model and uncorrelated lognormal molecular clock were assumed. As there are no fossil calibration points for southern African freshwater fishes, divergence times for lineages of the three genera were estimated under a uniform prior (using the ucld.mean) with a lower bound of 0.76 and an upper bound of 2.2% per million years. These values were chosen because they encompass the published range of cyt *b* substitution rates in teleost fishes [46], [59]–[62]. All other priors were set to default values. Analyses were run for 6 × 10^7^ generations and sampled every 1000 generations. The first 10 percent of the sampled trees was discarded as burn-in. Beast results were visualised using the program Tracer 1.5 [53] to assess the adequacy of the effective sample sizes (ESS) for each estimated parameter and to obtain the mean divergence estimates in millions of years and their 95 percent highest posterior density (HPD) for all nodes.

## Results

### *Galaxias* Lineage Diversity and Distribution

For the individuals of *Galaxias* analysed, the edited alignment of mtDNA cyt *b* sequences was 648 base pairs in length of which 183 sites were variable. These variable sites resulted in 74 unique haplotypes, and their geographic distribution is presented in Appendix S2.

**Figure 4:**
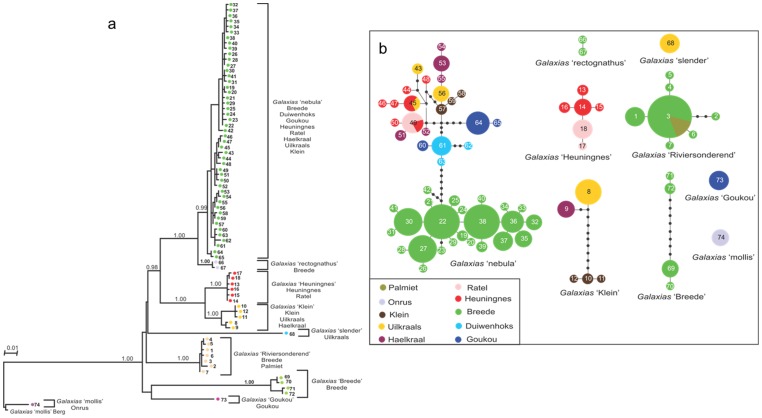
*Galaxias* lineage diversity. (a) Maximum Likelihood phylogenetic estimate of relationships among mitochondrial cytochrome *b* haplotypes of *Galaxias* from the south-western CFR. Bayesian posterior probabilities are given on the branches. The numbers (1–74) represent unique haplotypes and the colours indicate lineages, corresponding to distribution maps in Figure S1 in Appendix S2. River systems in which the lineages occur are listed below the lineage names and their ranges are presented in Figure S1 in Appendix S2. (b) TCS network of cyt *b* haplotypes (1–74) from individuals of *Galaxias* from the south-western CFR. The sizes of circles are proportional to haplotype frequency, and the colours indicate the river system(s) where the haplotype occurred. Black dots represent missing haplotypes in the network. Each branch represents one mutation step.

Tree topologies obtained by the BI and ML methods were identical, with only minor differences at some terminal nodes. The ML tree is presented with posterior probability support values from the BI analysis (Fig. 4a). Nine main clades (lineages) were found from the south-western CFR (Fig. 4a), with deep phylogenetic divergences among lineages (3.49–25.27%) (Table 1). Haplotypes were grouped within these lineages with high Bayesian posterior probability values (0.98–1.00) for those lineages that had multiple haplotypes. Deeper relationships among lineages were not well resolved, but *G.* sp. ‘rectognathus’ was resolved as sister to *G.* sp. ‘nebula’ (posterior probability = 1.00) and *G*. sp. ‘Heuningnes’ was resolved as sister to *G*. sp. ‘klein’ (posterior probability = 1.00). The clade containing *G*. sp. ‘Heuningnes’ and *G*. sp. ‘Klein’ was resolved as sister to the clade containing *G*. sp. ‘rectognathus’ and *G*. sp. ‘nebula’ (posterior probability = 0.98) (Fig. 4a). The TCS haplotype network (Fig. 4b) consisted of six distinct clades and three disconnected haplotypes, reflecting the same nine lineages as the BI and ML phylogenies.

**Table 1:** Means and ranges (in parentheses) of model-corrected genetic divergences (%) between *Galaxias* lineages from the south-western CFR.

	Lineage	1	2	3	4	5	6	7	8	9	*F*s
1	‘nebula’	**1.29 (0–2.56)**									−22.5**
2	‘rectognathus’	3.5 (2.4–4.3)	**0.16**								–
3	‘Heuningnes’	11.2(10.0–12.5)	11.04 (10.62–11.41)	**0.30 (0–0.31)**							−2.07
4	‘Klein’	11.46 (9.43–12.93)	10.67 (10.29–11.07)	4.40 (3.84–4.84)	**1.35 (0–2.06)**						3.32
5	‘slender'	17.71 (16.41–18.88)	17.15 (16.81–17.50)	18.23 (17.80–18.56)	17.07 (16.51–17.39)	**0.00**					–
6	‘Riviersonderend’	12.12 (10.91–13.90)	9.59 (9.03–10.32)	12.62 (11.41–14.11)	11.91 (10.50–13.36)	16.91 (16.49–17.96)	**0.38 (0–0.79)**				−3.14*
7	‘Breede’	18.24 (15.42–21.39)	18.76 (17.97–19.87)	18.56 (17.63–19.76)	17.79 (16.58–19.93)	24.86 (24.26–25.82)	18.02 (17.10–19.45)	**1.37 (0–1.86)**			–
8	‘Goukou'	13.72 (12.26–14.76)	13.89 (13.60–14.18)	12.45 (12.15–12.89)	13.22 (12.83–13.59)	17.61	11.37 (10.72–12.24)	16.65 (16.18–17.25)	**0.00**		**–**
9	‘mollis’	20.50 (18.85–22.51)	19.63 (19.23–20.03)	20.10 (19.83–20.48)	19.37 (18.51–19.92)	24.51	20.14 (19.56–21.19)	25.27 (24.19–26.81)	17.25	**0.00**	–

Within lineage divergences are shown in bold. Fu’s *F*s value for each lineage is given in the last column (** <0.005; *<0.05).

All the rivers mentioned in the text are presented in Fig. 1. Geographic distributions of the lineages are presented in Figure S1 in Appendix S2. *Galaxias* sp. ‘nebula’ is the most widespread of the nine lineages as it was collected in all river systems of the south-western CFR, except the Onrus and Palmiet Rivers. This lineage occurs in sympatry with six other lineages that have highly restricted geographic ranges (Fig. 4b; Figure S1 in Appendix S2). *Galaxias* sp. ‘Heuningnes’ was found in two currently isolated river systems, while *Galaxias* sp. ‘Klein’ was found in three river systems (Figure S1 in Appendix S2). *Galaxias* sp. ‘slender’ is restricted to the Uilkraals River system. *Galaxias* sp. ‘mollis’ occurs in the Onrus River system in the south-western CFR as well as in the Leeu River, a tributary of the Berg River system on the west coast. *Galaxias* sp. ‘Goukou’ was only found in the Goukou River system, while *Galaxias* sp. ‘rectognathus’ was collected in two tributaries of the Riviersonderend River, a major subcatchment of the Breede River system (Figure S1 in Appendix S2). *Galaxias* sp. ‘Riviersonderend’ was found in the upper Riviersonderend and six of its tributaries as well as in the Keurbooms River just below the confluence of the Breede and the Riviersonderend (Figure S1 in Appendix S2). This lineage was also collected in the Palmiet River system despite this system being separated from the Riviersonderend sub-catchment by the Hottentots Holland Mountain Range (Figure S1 in Appendix S2). *Galaxias* sp. ‘Breede’ was collected at three localities in the Breede River (Figure S1 in Appendix S2).

*Galaxias* sp. ‘nebula’ and *G*. sp. ‘Riviersonderend’ show significant signals of recent population expansion (Table 1). *Galaxias* sp. ‘Heuningnes’ had a negative *F*s value, but it was not statistically significant. *Galaxias* sp. ‘Klein’ showed no evidence of recent population expansion, while the other lineages had too few individuals for meaningful computation.

### Pseudobarbus Burchelli Lineage Diversity and Distribution

**Figure 5:**
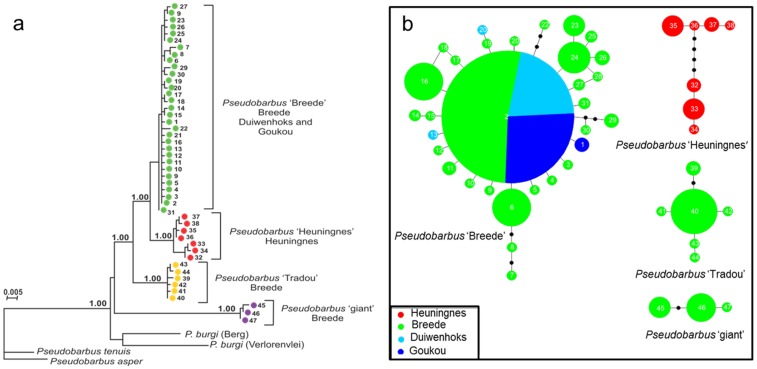
*Pseudobarbus* lineage diversity. (a) Maximum Likelihood analysis of phylogenetic relationships among mitochondrial cyt *b* haplotypes identified in *Pseudobarbus* from the south-western CFR. Bayesian posterior probabilities are given on the branches. The numbers (1–47) represent unique haplotypes and the colours represent unique lineages, corresponding to distribution maps in Figure S2 in Appendix S2. Distribution ranges of these lineages are presented in Figure S2 in Appendix S2. River systems in which the lineages occur are given in parentheses. (b) TCS network of cyt *b* haplotypes (1–47) from individuals of *Pseudobarbus* from the south-western CFR. The sizes of circles are proportional to haplotype frequency, and the colours indicate the river system (s) where the haplotype occurred. Black dots represent missing haplotypes in the network. Each branch represents one mutation step.

The edited alignment of mtDNA cyt *b* sequences from 177 individuals was 676 base-pairs long and 95 of these characters were variable. This resulted in 47 unique haplotypes. Both BI and ML analyses revealed identical relationships among historically-isolated lineages. The ML tree is presented with Bayesian posterior probabilities indicating branch support (Fig. 5a). The analyses revealed substantial genetic structuring within *P. burchelli*. Four distinct lineages: *Pseudobarbus* sp. ‘Breede’, *Pseudobarbus* sp. ‘Heuningnes’, *Pseudobarbus* sp. ‘Tradou’ and *Pseudobarbus* sp. ‘giant’ were identified (Fig. 5a). TCS analysis produced four distinct clades (Fig. 5b) reflecting the same lineages as the BI and ML phylogenies. Deep phylogenetic divergences were found among lineages (2.58–9.99%), while minor genetic divergences were found within lineages (0.30–0.71%) (Table 2).

**Table 2:** Mean and range of model-corrected genetic divergence (%) between *Pseudobarbus* lineages from the south-western CFR.

	Lineage	1	2	3	4	*F*s
1	‘Breede’	**0.43 (0–0.91)**				−33.50**
2	‘giant’	9.99 (9.25–10.97)	**0.30 (0–0.45)**			–
3	‘Tradou’	3.77 (3.20–4.27)	9.29 (8.79–9.98)	**0.33 (0–0.60)**		−2.18
4	‘Heuningnes’	2.58 (1.86–3.20)	9.92 (9.25–10.45)	4.75 (4.09–5.40)	**0.71 (0–1.38)**	0.93

The ranges of the values are given in parentheses. Within lineage divergences are given in bold. Fu’s *F*s value for each lineage is given in the last column (** <0.005; *<0.05).

*Pseudobarbus* sp. ‘Breede’, *P*. sp. ‘giant’ and *P*. sp. ‘Tradou’ are restricted to inland rivers, while P. sp. ‘Heuningnes’ was found exclusively at coastal sites. *Pseudobarbus* sp. ‘Breede’ is the most widespread of the four lineages, being distributed across the isolated Breede, Duiwenhoks and Goukou River systems (Figure S2 in Appendix S2). This lineage occurs in sympatry with P. sp. ‘giant’ in the upper Riviersonderend. *Pseudobarbus* sp. ‘Tradou’ is restricted to the Tradou River, a tributary of the Breede River system (Figure S2 in Appendix S2). *Pseudobarbus* sp. ‘Heuningnes’ is restricted to the Heuningnes River system (Figure S2 in Appendix S2). Results of Fu’s *F_s_* tests suggest recent population expansion for *Pseudobarbus* sp. ‘Breede’, while *P*. sp ‘Tradou’ and *P*. sp. ‘Heuningnes’ show no evidence of recent population expansion (Table 2).

### *Sandelia capensis* Lineage Diversity and Distribution

**Figure 6:**
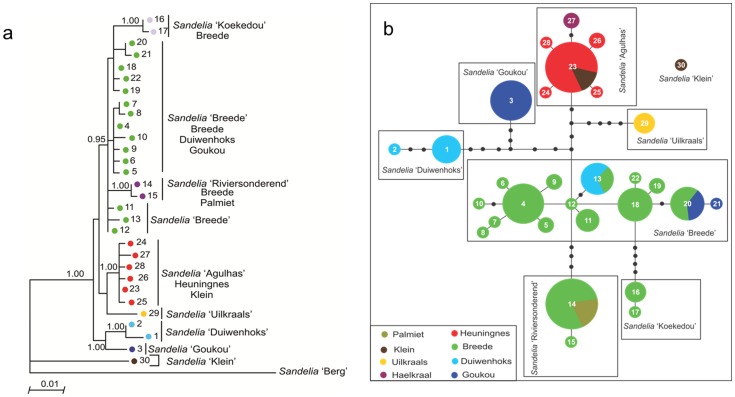
*Sandelia* lineage diversity. (a) Eight lineages with strong geographic affinities recovered with Maximum Likelihood phylogenetic analysis of mitochondrial cyt *b* haplotypes (1–30) identified in *Sandelia* from the south-western CFR. Bayesian posterior probabilities are given on the branches. The colours denote lineages and their distribution ranges are presented in Figure S3 in Appendix S2. (b) TCS network of cyt *b* haplotypes (1–30) from individuals of *Sandelia* from the south-western CFR. The sizes of circles are proportional to haplotype frequency, and the colours indicate the river system (s) where the haplotype occurred. Black dots represent missing haplotypes in the network. Each branch represents one mutation step.

DNA sequencing resulted in 98 sequences of the mtDNA cyt *b* gene and the edited alignment was 621 bp long. The sequences contained 66 variable sites which resulted in 30 haplotypes. BI and ML phylogenetic reconstructions (Fig. 6a) recovered eight lineages with strong geographical affinities (described below).

The TCS output consisted of one large network and a disconnected haplotype from the Klein River system (haplotype 30) (Fig. 6b). Within the large haplotype network, seven with strong geographical affinities can be identified. Similar to both *Galaxias* and *Pseudobarbus*, some *Sandelia* lineages are associated with coastal rivers while others are restricted to inland rivers. The first lineage, *Sandelia* sp. ‘Duiwenhoks’, comprised haplotypes that are restricted to the Duiwenhoks River system, and the second lineage, *Sandelia* sp. ‘Goukou’, occurred in the Goukou River system. The third lineage, *Sandelia* sp. ‘Breede’, comprised of haplotypes collected from multiple sites in the Breede, Duiwenhoks and Goukou River systems (Figure S3 in Appendix S2). The haplotypes which comprised the phylogenetically well-supported *Sandelia* sp. ‘Riviersonderend’ were restricted to the Riviersonderend and Palmiet Rivers (Figure S3 in Appendix S2). *Sandelia* sp. ‘Koekedou’, also well-supported in the tree, was restricted to localities in the upper Breede (Figure S3 in Appendix S2). *Sandelia* sp. ‘Agulhas’ (supported with a Bayesian posterior probability of 1.00) comprised of haplotypes from the Heuningnes, Haelkraal and Klein River systems, while *Sandelia* sp. ‘Uilkraals’ was restricted to the Uilkraals River system. Seven of the eight lineages had shallow divergences among them (1.03–2.86%), with *Sandelia* sp. ‘Klein’ being the only deeply divergent lineage within *Sandelia* (5.31–6.09%; Table 3).

**Table 3:** Mean and range of model-corrected genetic distances between eight lineages identified within *Sandelia* from the south-western CFR.

		i	ii	iii	iv	v	vi	vii	Klein	*F*s
i	‘Duiwenhoks’	**0.49**								–
ii	‘Goukou’	1.43 (1.16–1.70)	**0.00**							–
iii	‘Breede’	1.94 (1.35–2.66)	1.56 (1.18–1.91)	**0.61 (0.16–1.38)**						−4.97**
iv	‘Riviersonderend’	2.09 (1.34–2.86)	1.82 (1.52–2.10)	1.03 (0.83–1.18)	**0.16**					**–**
v	‘Koekedou’	2.86 (2.46–3.27)	2.40 (2.30–2.49)	1.45 (1.01–1.91)	1.72 (1.35–2.10)	**0.16**				**–**
vi	‘Agulhas’	1.92 (1.52–2.31)	1.84 (1.70–1.93)	1.35 (0.83–1.89)	1.62 (1.16–2.08)	2.19 (1.90–2.47)	**0.33 (0.16–0.50)**			−2.57*
vii	‘Uilkraals’	1.99 (1.71–2.27)	2.26	1.71 (1.35–2.11)	1.98 (1.69–2.27)	2.56 (2.46–2.66)	1.35 (1.17–1.53)	**0.00**		**–**
	‘Klein’	6.09 (5.72–6.47)	5.57	5.31 (4.90–5.94)	5.47 (5.30–5.65)	6.00 (5.83–6.08)	5.72 (5.53–5.98)	5.72	**0.00**	**–**

Within lineage divergences are given in bold. Fu’s *F*s value for each lineage are given in the last column (** <0.005; *<0.05).

Fu’s *F*_s_ statistics were only computed for the ’Breede’ and ‘Agulhas’ lineages of *Sandelia*. Recent population expansion was detected for both lineages (Table 3). The numbers of the other lineages were too low for reliable computation of neutrality statistics.

### Estimates of Divergence Times

**Figure 7:**
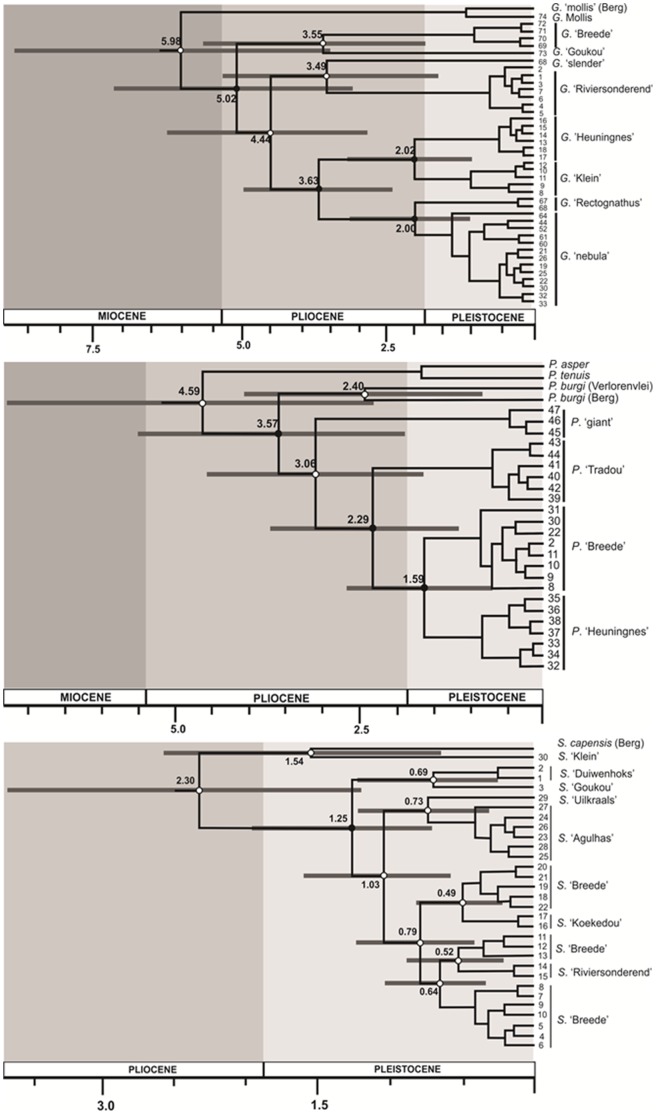
Chronograms with estimates of divergence times (Million years ago). Values on the nodes represent the estimated mean divergence dates for major lineages of (a) *Galaxias*, (b) *Pseudobarbus* and (c) *Sandelia* inferred using Bayesian coalescent analyses implemented in BEAST. Bars represent 95% highest posterior densities for divergence estimates. Solid circles represent posterior probability values greater or equal to 0.95 and open circles represent values less than 0.95.

Estimates of divergence times for the main lineages of *Galaxias*, *Pseudobarbus* and *Sandelia* are shown in chronograms in Fig 7. Cladogenesis in *Galaxias* and *Pseudobarbus* largely occurred within a period bounded by the Late Miocene-Early Pliocene (Fig 7a,b), a period characterised by increased sea-levels. Dating estimates suggested that the deepest split in *Sandelia* occurred at the end of the Pliocene, but much of the phylogeographic structuring in this genus occurred during the Pleistocene (Fig 7c).

## Discussion

The study uncovered nine deeply-divergent lineages of *Galaxias*, four historically-isolated lineages of *Pseudobarbus* and at least two deeply-divergent lineages of *Sandelia* from the south-western CFR. All the lineages (except *Galaxias* sp ‘nebula’ and *Galaxias* sp ‘mollis’) are restricted to the south-western CFR, and we have presented in the present study their entire known ranges. Taxonomic statuses of newly identified lineages from DNA-based studies are being confirmed with additional analyses, including morphology. These results thus complement previous studies [22], [43], [44], [63] providing strong evidence that the south-western CFR represents a previously unrecognized centre of stream fish diversity and endemism in the broader CFR. The goal of the present study was to investigate the mechanisms that drove lineage diversification and shaped the geographical distribution patterns of this assemblage of stream fishes by testing three hypotheses: Refugia, Palaeoriver and Inter-drainage dispersal.

### Refugia Hypothesis

Accumulating evidence indicates that fragmentation of populations into separate refugia is an important mechanism that drove lineage splitting in several primary freshwater fishes [45], [64]–[68]. Results of the present study support the Refugia hypothesis. Divergence time estimates indicate that splitting of major lineages within *Galaxias*, *Pseudobarbus* and *Sandelia* coincided with a period of higher sea-levels, suggesting that fragmentation of populations of these taxa in upland refugia promoted diversification. Many of the deeply divergent lineages have strong geographical affinities, with distribution ranges restricted to specific river systems that were not completely inundated during the Miocene-Pliocene marine transgression. For example, restriction of the deeply-divergent lineage, *Sandelia* sp. ‘Klein’, to the Klein River system is consistent with the expectation that the upper reaches of the Klein provided important refuge to freshwater taxa during the last major sea-level transgression. Similarly, restriction of *Galaxias* sp. ‘slender’ and *Galaxias* sp. ‘Goukou’ to the Uilkraals and Goukou River systems, respectively, is evidence that both river systems served as important refugia for freshwater fishes during the Miocene-Pliocene high sea-levels. The distribution limits of *Galaxias* sp. ‘Riviersonderend’ and *Galaxias* sp. ‘Breede’ suggest that these lineages could have evolved in allopatry due to vicariance caused by possible isolation of the Breede and Riviersonderend catchments during the Miocene-Pliocene marine incursion. Similarly, *Pseudobarbus* sp. ‘Breede’, *Pseudobarbus* sp. ‘Tradou’ and *Pseudobarbus* sp. ‘giant’ could have diverged through allopatric isolation due to possible vicariance of the Breede, Riviersonderend and Tradou Rivers, followed by post-speciation dispersal for *Pseudobarbus* sp. ‘Breede’ and *Pseudobarbus* sp. ‘giant’ to attain their present day distributions. These results are consistent with findings from other regions where marine incursions are considered to have isolated and drove diversification of freshwater taxa [45], [64]–[66].

### Palaeoriver Hypothesis

The Miocene-Pliocene transgression was followed by a major regression during the last glacial maximum (LGM) [30], resulting in the confluence of several adjacent rivers before reaching the sea [8], [13], [14]. The palaeorivers of the LGM have been proposed as a plausible explanation for the common occurrence of *Pseudobarbus* lineages across currently-isolated river systems in the CFR [13], [14], [63]. Interconnections of rivers following exposure of the continental shelf during glacial periods [69], [70] have also been inferred to have facilitated dispersal of *Percicthys trucha*, a Patagonian freshwater fish with an extensive geographical range, but lacking phylogeographic structuring [71].

Results from the present study show some support for the Palaeoriver hypothesis. Occurrence of *Pseudobarbus* sp. ‘Breede’ and *Sandelia* sp. ‘Breede’ in the currently isolated Breede and Duiwenhoks River systems is consistent with the confluence of these rivers during the LGM low sea-levels. However, occurrence of these lineages in the Goukou River system is not consistent with the Palaeoriver hypothesis, because the Goukou was part of an eastern palaeoriver system (the Gouritz-Goukou). This therefore suggests the role of alternative mechanisms (see below).

The lack of sharing of lineages (except *Galaxias* sp. ‘nebula’) among the Breede, Duiwenhoks and Heuningnes was also surprising, because these river systems coalesced as recently as the LGM. This is perhaps a result of the extreme hydrochemical differences between inland and coastal draining river systems in the south-western CFR. Most tributaries of inland draining rivers (including the Breede and Duiwenhoks river systems) have clear, oligotrophic water with low conductivity, while the coastal-draining rivers on the Agulhas Plain (including the Heuningnes River system) have tannin stained ‘brown’ water with high conductivity due to high salt content [38]. We hypothesise that the extreme ecological gradient between the Heuningnes and the inland rivers (Breede and Duiwenhoks) may represent a physiological barrier that could have hampered exchange of lineages between these systems. This is assuming that the current ecological differences were present and persisted when the river systems shared a common confluence. This hypothesis of the role of ecological gradients is corroborated by recent findings for Amazonian fishes where extreme contrast in optical characteristics of riverine waters has been a major driver of ecological divergence, speciation and distribution of cryptic lineages and species [6], [72], [73].

### Inter-drainage Dispersal Hypothesis

*Galaxias* sp. ‘Heuningnes’ shows no differentiation between the Heuningnes and Ratel River systems despite these systems being currently isolated. As the entire Ratel River system was under marine water during the Miocene-Pliocene transgression, it is likely that freshwater taxa in this river system were extirpated. Freshwater taxa in the Ratel are therefore likely to be recent immigrants from adjacent refugial populations. *Galaxias* sp. ‘Heuningnes’ is likely to have survived and evolved in the Heuningnes River system and then dispersed to the Ratel River system. Since the Ratel and Heuningnes did not share a common confluence during the LGM, dispersal via intermittent freshwater connections is the most plausible explanation for the lack of differentiation between these two river systems. The lack of a discernible drainage divide between the Ratel and a western tributary of the Heuningnes could have allowed movement following episodic connections during periods of heavy flooding.

Similarly, *Galaxias* sp. ‘Klein’ has closely related haplotypes across the Klein, Uilkraals and Haelkraal, despite the current hydrological isolation of these river systems. These river systems did not coalesce during the LGM low sea-levels. Overland dispersal via intermittent freshwater connections during pluvial periods is therefore the most likely explanation for the current distribution of *Galaxias* sp. ‘Klein’. Since the Haelkraal is likely to have been submerged during the Miocene-Pliocene transgression, *Galaxias* sp. ‘Klein’ is likely to have survived in either the Klein or the Uilkraals. The restriction of the genetically-distinct *Galaxias* sp. ‘slender’ to the Uilkraals and the relatively smaller size of this river system suggests that *Galaxias* sp. ‘Klein’ and *Galaxias* sp. ‘slender’ are likely to have evolved in allopatry, and their co-occurrence in the Uilkraals could be a result of secondary contact. It is therefore logical to suggest that *Galaxias* sp. ‘Klein’ could have evolved in the Klein River system due to possible vicariant isolation by the middle Miocene to early Pliocene marine incursions. This would then be consistent with the *Sandelia* sp. ‘Klein’ scenario discussed earlier.

*Sandelia* sp. ‘Agulhas’ also occurs across several currently isolated river systems that did not coalesce during the LGM regression. Low genetic differentiation between *Sandelia* sp. ‘Agulhas’ across the Heuningnes, Haelkraal, Uilkraals and Klein Rivers suggests either recent disruption of gene flow or recent range expansion. Historical panmixia is unlikely, particularly given the historical oscillations between extremes of dry and wet conditions that were experienced in southern Africa [36]. A more plausible explanation is, therefore, that *Sandelia* sp. ‘Agulhas’ could have evolved in isolation and only recently expanded its range across river systems draining the Agulhas Plain. Based on the existence of distinct lineages of *Sandelia* in the Klein and Uilkraals, and the fact that the Haelkraal was drowned during the Miocene-Pliocene transgression, it is more likely that *Sandelia* sp. ‘Agulhas’ evolved in the Heuningnes River system. The low genetic differentiation in this lineage among these river systems may be evidence for more recent dispersal via low drainage divides during wet climatic periods. This mechanism was used to explain the wide geographic range of *Galaxias* sp. ‘nebula’ across the CFR [8]. Thus, the sympatric occurrence of two lineages of *Sandelia* in the Uilkraals and the Klein could be a result of secondary contact due to post-speciation dispersal of *Sandelia* sp. ‘Agulhas’.

The occurrence of *Pseudobarbus* sp. ‘Breede’ and *Sandelia* sp. ‘Breede’ in the Goukou River system is not consistent with the sea-level regression hypothesis because the Goukou belonged to a different palaeoriver system. This suggests the role of alternative dispersal mechanisms such as a rare river capture event or episodic connection possibly during inundation of low drainage divides during wetter climatic periods. The upper Duiwenhoks and the Korinte River (a tributary of the Goukou) are separated by a low drainage divide which could have allowed interdrainage movement during pluvial periods. Partridge *et al*., [34] inferred wetter climatic conditions for coastal areas of the southern Cape occurring as recently as the Holocene Altithermal (*ca*. 8 000–6 000 years ago). Connections of low drainage divides during wetter climatic periods have been proposed to have facilitated interdrainage movement of obligate freshwater taxa in the CFR [8] and elsewhere [74], [75].

Human-mediated translocations could have also influenced the distribution patterns of *Sandelia* sp. ‘Breede’ and *Pseudobarbus* sp. ‘Breede’. Chakona & Swartz [76] reported that *Sandelia* lineages in undisturbed tributaries of the Breede River system are restricted to river reaches below 425 m elevation, while *Galaxias* sp. ‘nebula’ and *Pseudobarbus* sp. ‘Breede’ always occurred at higher elevations than the *Sandelia* lineages. This pattern is reversed in the Goukou, with *Sandelia* sp. ‘Breede’ occurring above major waterfalls that are effective barriers for both *Galaxias* sp. ‘nebula’ and *Pseudobarbus* sp. ‘Breede’. This and the fact that individuals of *Sandelia* sp. ‘Breede’ collected from populations above one of the major waterfalls in the Goukou contained Breede haplotypes suggests that *Sandelia* sp. ‘Breede’ in the Goukou could have been introduced from the Breede River system. Since *Sandelia* sp. ‘Breede’ and *Pseudobarbus* sp. ‘Breede’ co-occur in large numbers within the Breede, it is possible that they could have both been introduced into the Goukou River system. This hypothesis is subject for further investigation with additional markers such as microsatellites that will help to discriminate between natural and human aided movements. The occurrence of *Galaxias* sp. ‘Riviersonderend’ and *Sandelia* sp. ‘Riviersonderend’ in the Palmiet River system was also unexpected because the Riviersonderend and Palmiet are separated by a high mountain barrier. This suggests possible human-mediated translocation that also warrants further investigation.

### Test for Shared Evolutionary History

*Galaxias*, *Pseudobarbus* and *Sandelia* show broad similarities in the distribution of lineages across the south-western CFR because they all have: (1) genetically-distinct lineages restricted to the coastal river systems of the Agulhas Plain and inland lineages restricted to the Breede, Duiwenhoks and Goukou River systems, (2) some lineages that show low or no differentiation across currently isolated river systems and (3) deeply divergent lineages that occur in sympatry. *Galaxias* and *Sandelia*, in particular, each have distinct lineages that largely co-occur. For example, they both have distinct lineages that are restricted to the Goukou, Riviersonderend and Palmiet, Uilkraals and Klein. Common phylogeographic patterns in multiple sympatric species across a wide geographic range may suggest similar responses to common regional scale physical processes [45], [77]. However, for patterns to maximally represent simultaneous responses to evolutionary forces, they must also be temporally concordant [78]. Temporal patterns of divergence between *Galaxias* and *Sandelia* are, however, not consistent with spatial patterns because the species showed substantial differences in the timing of cladogenesis. This may suggest the influence of similar evolutionary forces, such as historical isolation followed by subsequent range expansion, but these events could have affected the two genera at different time periods.

Alternatively, the lack of temporal concordance between *Sandelia* compared to *Galaxias* and *Pseudobarbus* could be a result of differences in the mutation rate of their mitochondrial DNA. Significant differences in the rates of molecular evolution have also been reported among the anabantoid fishes [1], which include the genus *Sandelia*. This presents an interesting case that warrants further investigation.

## Conclusions

The results of the present study are consistent with palaeogeographic hypotheses invoked to explain phylogeographic patterns from other regions of the world [45], [64]–[66]. These studies provided evidence that sea-level fluctuations played a major role in driving diversification and influencing biogeographic patterns of extant freshwater taxa in the Neotropics [45], [66], North America [64] and the Indo-Australian Archipelago [65]. Along similar lines, diversification of freshwater fishes within the south-western CFR appears to have been generated predominantly by passive vicariance of river systems during the Miocene-Pliocene transgression. Coalescence of adjacent rivers due to the subsequent sea-level regression during the LGM facilitated postspeciation range expansion (dispersal) of some lineages to colonise currently isolated river systems. Dispersal could have also been facilitated by rare events such as intermittent wet connections during pluvial periods [8]. Thus, the sympatric occurrence of deeply divergent lineages in each of the three genera could be a result of secondary contact through dispersal from Miocene-Pliocene refugial populations. This paleogeographic and dispersal hypothesis for freshwater fishes of the south-western CFR is summarised in Figures S1–S3 in Appendix S3. Future studies that incorporate multiple genetic markers, as well as other freshwater-restricted taxa (for example macroinvertebrate groups with low dispersal abilities), are encouraged to further test and refine the hypotheses presented in the present study regarding the diversification and biogeographical patterns of freshwater taxa in this global biodiversity hotspot.

## Supporting Information

Appendix S1 **Sampling localities in the south-western Cape Floristic Region of South Africa.** Tributaries sampled for *Galaxias* (*Gal*), *Pseudobarbus* (*Pse*) and *Sandelia* (*San*) from the south-western CFR. Locality codes and geographic coordinates are given. The number of individuals that were sequenced per locality is indicated. Blank space indicates that individuals of that genus were not collected at that locality.
(DOCX)

Appendix S2 **Distribution of** ***Galaxias*** **,** ***Pseudobarbus*** **and** ***Sandelia*** **lineages in the south-western Cape Floristic Region of South Africa.**
(DOCX)

Appendix S3 **Conceptual models for diversification and geographical distribution.** Hypothesised processes that drove diversification and shaped the distribution of unique lineages within *Galaxias*, *Pseudobarbus* and *Sandelia* in the south-western Cape Floristic Region of South Africa.
(DOCX)
