# Supplementary material for: Evolutionary Drivers of Diversification and Distribution of a Southern Temperate Stream Fish Assemblage: Testing the Role of Historical Isolation and Spatial Range Expansion
Source: PLoS One. 2013 Aug 9;8(8):e70953. doi: 10.1371/journal.pone.0070953 (PMC3739774; doi:10.1371/journal.pone.0070953)
Supplement: Appendix S1 — Sampling localities in the south-western Cape Floristic Region of South Africa. Tributaries sampled for Galaxias (Gal), Pseudobarbus (Pse) and Sandelia (San) from the south-western CFR. Locality codes and geographic coordinates are given. The number of individuals that were sequenced per locality is indicated. Blank space indicates that individuals of that genus were not collected at that locality. (DOCX) [file pone.0070953.s001.docx]

**SUPPORTING INFORMATION**

**Evolutionary drivers of diversification and distribution of a southern temperate stream fish assemblage: testing the role of historical isolation and spatial range expansion**

Albert Chakona, Ernst R. Swartz and Gavin Gouws

**Appendix S1** **Sampling localities in the south-western Cape Floristic Region of South Africa.** Tributaries sampled for *Galaxias* (*Gal*), *Pseudobarbus* (*Pse*) and *Sandelia* (*San*) from the south-western CFR. Locality codes and geographic coordinates are given. The number of individuals that were sequenced per locality is indicated. Blank space indicates that individuals of that genus were not collected at that locality.

|  | Sample Code | River | System | Latitude | Longitude | *Gal* | *Pse* | *San* |
| --- | --- | --- | --- | --- | --- | --- | --- | --- |
| 1 | AC08A07 | Vink | Breede | -33.73422222 | 19.82030556 |  | 2 |  |
| 2 | AC08A10 | Vink | Breede | -33.73022222 | 19.82355556 | 2 |  |  |
| 3 | AC08A11 | Noree | Breede | -33.72494444 | 19.81547222 | 1 |  |  |
| 4 | AC08A12 | Hoeks | Breede | -34.02316667 | 19.83852778 |  |  | 1 |
| 5 | AC08A13 | Hoeks | Breede | -34.01091667 | 19.83922222 | 1 |  | 1 |
| 6 | AC08A14 | Hoeks | Breede | -34.00963889 | 19.83836111 | 1 | 2 |  |
| 7 | AC08A17 | Noree | Breede | -33.73277778 | 19.81361111 |  | 2 |  |
| 8 | AC08A18 | Hex | Breede | -33.49877778 | 19.53061111 | 2 |  |  |
| 9 | AC08A19 | Hex | Breede | -33.52894444 | 19.54008333 | 3 | 2 | 2 |
| 10 | AC08A20 | Amandel | Breede | -33.52402778 | 19.54266667 | 2 | 2 |  |
| 11 | AC08A21 | Amandel | Breede | -33.50661111 | 19.49497222 |  |  | 1 |
| 12 | AC08A31 | Amandel | Breede | -33.99380556 | 19.18263889 |  | 2 |  |
| 13 | AC08A32 | Amandel | Breede | -33.98900000 | 19.18402778 | 2 |  | 2 |
| 14 | AC08A38 | Amandel | Breede | -33.97447222 | 19.19175000 | 3 | 2 |  |
| 15 | AC08A42 | Amandel | Breede | -33.98297222 | 19.18963889 | 2 |  |  |
| 16 | AC08A52 | Wit | Breede | -33.57322222 | 19.13797222 |  | 2 | 2 |
| 17 | AC09A08 | Tradou | Breede | -33.95677778 | 20.70788889 |  | 1 |  |
| 18 | AC09A09 | Tradou | Breede | -33.94155556 | 20.70733333 |  | 2 |  |
| 19 | AC09A42 | Wolwekloof | Breede | -33.56413889 | 19.12933333 | 4 |  |  |
| 20 | AC09A43 | Wolwekloof | Breede | -33.56505556 | 19.13144444 |  | 2 | 2 |
| 21 | AC09A46 | Titus | Breede | -33.40533333 | 19.41805556 |  | 2 | 2 |
| 22 | AC09A50 | Keissies | Breede | -33.71050000 | 19.93105556 | 2 |  |  |
| 23 | AC09A51 | Keissies | Breede | -33.70352778 | 19.91086111 | 3 |  |  |
| 24 | AC09A54 | Keissies | Breede | -33.71050000 | 19.93105556 | 1 |  |  |
| 25 | AC09A58 | Koo | Breede | -33.59450000 | 19.75811111 |  | 2 | 2 |
| 26 | AC09A61 | Die Brak | Breede | -33.57644444 | 19.75163889 |  | 2 |  |
| 27 | AC09A63 | Elandskloof | Breede | -34.03055556 | 19.39000000 |  | 2 |  |
| 28 | AC09A64 | Elandskloof | Breede | -34.03461111 | 19.39327778 |  | 2 |  |
| 29 | AC09A65 | Baviaans | Breede | -34.02705556 | 19.55588889 |  | 2 | 2 |
| 30 | AC09A70 | Gobos | Breede | -34.03661111 | 19.63613889 | 2 | 2 | 2 |
| 31 | AC09A71 | Gobos | Breede | -34.03833333 | 19.62844444 | 1 |  |  |
| 32 | AC09A72 | Gobos | Breede | -34.03638889 | 19.62575000 | 1 | 2 |  |
| 33 | AC09A73 | Ganskraal | Breede | -34.11577778 | 19.81394444 |  | 2 | 1 |
| 34 | AC09A74 | Happy Valley | Breede | -34.11183333 | 19.81530556 | 2 | 2 |  |
| 35 | AC09A75 | Happy Valley | Breede | -34.11222222 | 19.69797222 | 2 |  | 1 |
| 36 | AC09A76 | Ganskraal | Breede | -34.12547222 | 19.81147222 | 2 |  |  |
| 37 | AC09A78 | Boks | Breede | -34.07963889 | 19.82952778 | 4 |  |  |
| 38 | AC09A79 | Boks | Breede | -34.09602778 | 19.82841667 |  | 2 |  |
| 39 | AC09B14 | Boesmans | Breede | -34.01113889 | 20.00722222 |  | 1 |  |
| 40 | AC09B15 | Boesmans | Breede | -34.05100000 | 19.95300000 |  |  | 2 |

Appendix S1: continued...

|  | Sample Code | River | System | Latitude | Longitude | *Gal* | *Pse* | *San* |
| --- | --- | --- | --- | --- | --- | --- | --- | --- |
| 41 | AC09B17 | Boesmans | Breede | -34.04158333 | 19.96205556 |  | 1 |  |
| 42 | AC09B18 | Poesjenels | Breede | -33.96547222 | 19.58336111 |  |  | 2 |
| 43 | AC09B20 | Poesjenels | Breede | -33.97916667 | 19.55761111 | 1 |  |  |
| 44 | AC09B22 | Dorn | Breede | -33.85333333 | 19.47580556 |  |  | 1 |
| 45 | AC09B23 | Riesvlei | Breede | -33.89316667 | 19.44344444 |  |  | 1 |
| 46 | AC09B25 | Dorn | Breede | -33.93294444 | 19.46161111 | 1 |  |  |
| 47 | AC09B27 | Stettyn | Breede | -33.86977778 | 19.34750000 | 1 |  |  |
| 48 | AC09B30 | Stettyn | Breede | -33.86847222 | 19.34725000 |  | 2 |  |
| 49 | AC09B33 | Holsloot | Breede | -33.82883333 | 19.27161111 | 2 |  | 2 |
| 50 | AC09B35 | Agter-Vink | Breede | -33.67155556 | 19.74255556 | 2 |  |  |
| 51 | AC09B38 | Spreeudrifspruit | Breede | -33.98316667 | 19.32925000 |  | 2 | 2 |
| 52 | AC09B39 | Bereaville | Breede | -34.05930556 | 19.47736111 |  |  | 2 |
| 53 | AC09B41 | Bereaville | Breede | -34.02705556 | 19.48236111 | 5 |  |  |
| 54 | AC09B42 | Bereaville | Breede | -34.03036111 | 19.48377778 |  | 2 |  |
| 55 | AC09B43 | Riviersonderend | Breede | -34.06227778 | 19.07013889 |  | 2 |  |
| 56 | AC09B44 | Riviersonderend | Breede | -34.06741667 | 19.07566667 | 4 |  |  |
| 57 | AC09B46 |  | Breede | -34.05486111 | 19.09744444 | 2 |  |  |
| 58 | AC09C25 |  | Breede | -34.37052778 | 20.64566667 |  | 4 | 1 |
| 59 | AC09C26 |  | Breede | -34.38255556 | 20.67505556 |  |  | 1 |
| 60 | AC09C27 |  | Breede | -34.38694444 | 20.68622222 |  |  | 1 |
| 61 | AC09C56 |  | Breede | -34.04236111 | 19.53716667 | 2 |  |  |
| 62 | AC09C57 |  | Breede | -34.07666667 | 19.64325000 | 2 |  |  |
| 63 | AC09C59 | Gobos | Breede | -34.05344444 | 19.61366667 | 2 |  |  |
| 64 | AC09C61 |  | Breede | -34.06538889 | 19.65883333 | 2 |  | 2 |
| 65 | AC09C63 | Keurbooms | Breede | -34.00441667 | 20.37713889 | 4 |  |  |
| 66 | AC09C65 | Soetmelks | Breede | -34.11238889 | 19.74552778 | 2 |  |  |
| 67 | AC09C66 | Soetmelksvlei | Breede | -34.10330556 | 19.74122222 |  | 2 |  |
| 68 | AC09C67 | Krom | Breede | -34.11166667 | 19.79566667 | 1 | 2 |  |
| 69 | AC09C69 | Klip | Breede | -34.02463889 | 20.41805556 | 2 | 2 | 2 |
| 70 | AC09C71 | Leeu | Breede | -33.97855556 | 20.34202778 | 1 |  |  |
| 71 | AC09C72 | Leeu | Breede | -33.99508333 | 20.33719444 |  | 2 |  |
| 72 | AC09C74 | Keurbooms | Breede | -33.98561111 | 20.37300000 |  | 3 |  |
| 73 | AC09C75 | Keurbooms | Breede | -33.99644444 | 20.37375000 | 2 |  |  |
| 74 | AC09C77 | Koornings | Breede | -34.01230556 | 20.45458333 | 2 | 1 | 2 |
| 75 | AC09C79 | Hartbees | Breede | -34.01230556 | 20.45458333 | 3 | 1 |  |
| 76 | AC09C81 | Bothaspruit | Breede | -33.56247222 | 19.34075000 | 2 | 5 | 1 |
| 77 | AC09C82 | Du Toit | Breede | -33.96702778 | 19.16566667 | 4 |  | 2 |
| 78 | AC09C84 | Slanghoek | Breede | -33.61155556 | 19.22402778 | 2 |  |  |
| 79 | AC09C85 | Slanghoek | Breede | -33.65522222 | 19.21797222 |  |  | 1 |
| 80 | AC09D08 | Breede | Breede | -33.68705556 | 19.42683333 | 4 |  |  |

Appendix S1: continued…

|  | Sample Code | River | System | Latitude | Longitude | *Gal* | *Pse* | *San* |
| --- | --- | --- | --- | --- | --- | --- | --- | --- |
| 81 | AC09D09 | Breede | Breede | -33.52002778 | 19.18552778 | 2 |  |  |
| 82 | AC09D16 | Du Toit | Breede | -33.93722222 | 19.16800000 |  | 4 |  |
| 83 | AC09D18 | Bothaspruit | Breede | -33.59952778 | 19.32330556 |  | 1 |  |
| 84 | AC09D20 | Wabooms | Breede | -33.50252778 | 19.27063889 | 2 |  |  |
| 85 | AC09D23 | Koekedou | Breede | -33.35911111 | 19.29547222 |  |  | 2 |
| 86 | AC09D25 | Wit tributary | Breede | -33.53744444 | 19.14302778 |  | 2 |  |
| 87 | AC09D28 | Krom | Breede | -33.72277778 | 19.11294444 |  | 6 |  |
| 88 | AC09D32 | Smallblaar | Breede | -33.69050000 | 19.31622222 | 2 |  |  |
| 89 | AC09D33 | Slanghoek | Breede | -33.66619444 | 19.24638889 | 2 |  |  |
| 90 | AC09D34 | Riviersonderend | Breede | -34.06230556 | 19.07050000 |  | 8 |  |
| 91 | AC09D39 | Sandrifs Dam | Breede | -33.43613889 | 19.57088889 |  |  | 2 |
| 92 | AC09D41 | Buffeljags | Breede | -33.99916667 | 20.60455556 | 2 |  |  |
| 93 | ES07A21 | Baviaans | Breede | -34.02705556 | 19.55588889 | 2 |  |  |
| 94 | gz32 | Happy Valley | Breede | -34.09650000 | 19.73605556 | 1 |  |  |
| 95 | gz33 | Elandskloof | Breede | -34.05202778 | 19.41627778 | 1 |  |  |
| 96 | AC09B13 | Duiwenhoks | Duiwenhoks | -33.99766667 | 21.10583333 |  |  | 2 |
| 97 | AC09C32 | Duiwenhoks | Duiwenhoks | -33.99480556 | 21.07519444 | 2 |  |  |
| 98 | AC09C33 | Duiwenhoks | Duiwenhoks | -33.97819444 | 21.03244444 | 2 |  | 2 |
| 99 | AC09C35 | Duiwenhoks | Duiwenhoks | -33.97041667 | 20.98783333 | 2 |  | 2 |
| 100 | AC09C36 | Duiwenhoks | Duiwenhoks | -33.99211111 | 20.85430556 | 1 |  | 2 |
| 101 | AC09C38 | Duiwenhoks | Duiwenhoks | -34.00705556 | 20.88383333 |  | 8 |  |
| 102 | AC09C39 | Duiwenhoks | Duiwenhoks | -34.01963889 | 20.93455556 |  | 4 | 2 |
| 103 | AC08A58 | Vette | Goukou | -34.02125000 | 21.22500000 | 4 | 2 | 2 |
| 104 | AC09B01 | Goukou | Goukou | -34.00036111 | 21.41800000 | 7 |  | 2 |
| 105 | AC09B04 | Kleinkruis | Goukou | -34.01719444 | 21.29058333 | 2 | 2 |  |
| 106 | AC09B06 | Kleinkruis | Goukou | -33.99900000 | 21.29138889 |  |  | 2 |
| 107 | AC09B07 | Kleinkruis | Goukou | -34.00272222 | 21.29222222 |  | 4 |  |
| 108 | AC09B10 | Kleinkruis | Goukou | -33.96108333 | 21.30677778 |  |  | 2 |
| 109 | AC09B11 | Vette | Goukou | -33.94430556 | 21.25419444 |  |  | 2 |
| 110 | AC09B12 | Vette | Goukou | -33.98491667 | 21.20716667 |  | 8 |  |
| 111 | AC09C31 | Korinte | Goukou | -33.98883333 | 21.15794444 | 2 |  | 2 |
| 112 | AC09A28 | Haelkraal | Haelkraal | -34.66763889 | 19.57527778 | 3 |  |  |
| 113 | AC09A29 | Haelkraal | Haelkraal | -34.66775000 | 19.57791667 |  |  | 2 |
| 114 | AC09A30 | Haelkraal | Haelkraal | -34.67683333 | 19.58697222 | 6 |  |  |
| 115 | ES07A38 | Haelkraal | Haelkraal | -34.67083333 | 19.54300000 | 2 |  |  |
| 116 | AC08A22 | Kars | Heuningnes | -34.41316667 | 19.82041667 | 2 | 2 |  |
| 117 | AC08A23 | Kars | Heuningnes | -34.41330556 | 19.82080556 |  |  | 4 |
| 118 | AC08A24 | Kars | Heuningnes | -34.36372222 | 19.78255556 |  | 2 | 1 |
| 119 | AC08A25 | Kars | Heuningnes | -34.36855556 | 19.78308333 |  |  | 1 |
| 120 | AC09A23 | Grashoek | Heuningnes | -34.58238889 | 19.96841667 |  |  | 2 |

Appendix S1: continued…

|  | Sample Code | River | System | Latitude | Longitude | *Gal* | *Pse* | *San* |
| --- | --- | --- | --- | --- | --- | --- | --- | --- |
| 121 | AC09A24 | Grashoek | Heuningnes | -34.57400000 | 19.94888889 | 1 | 3 |  |
| 122 | AC09A25 | Nuwejaars | Heuningnes | -34.55497222 | 19.88583333 | 1 |  |  |
| 123 | AC09A26 | Nuwejaars | Heuningnes | -34.54230556 | 19.81916667 | 2 |  | 2 |
| 124 | AC09A27 | Nuwejaars | Heuningnes | -34.57836111 | 19.75758333 | 3 | 4 | 2 |
| 125 | AC09B51 | Van Zyl farm tributary | Heuningnes | -34.51836111 | 19.97591667 | 1 |  |  |
| 126 | AC09C12 | Koue | Heuningnes | -34.56136111 | 19.63094444 | 2 |  |  |
| 127 | AC09C15 | Kars | Heuningnes | -34.46783333 | 19.82311111 | 2 |  | 1 |
| 128 | AC09C16 | Kars | Heuningnes | -34.45166667 | 19.90116667 | 3 |  | 2 |
| 129 | AC09C17 | Kars | Heuningnes | -34.41677778 | 19.80119444 |  | 1 |  |
| 130 | AC09B50 | Klein | Klein | -34.27619444 | 19.52513889 |  |  | 2 |
| 131 | AC09C05 | Hartbees | Klein | -34.32511111 | 19.37447222 | 4 |  |  |
| 132 | AC09C07 | Hartbees | Klein | -34.33419444 | 19.35850000 | 4 |  | 1 |
| 133 | ES07A42 | Onrus | Onrus | -34.37825000 | 19.24000000 | 4 |  |  |
| 134 | AC09B49 | Palmiet | Palmiet | -34.06919444 | 19.05072222 |  |  | 2 |
| 135 | AC09B55 | Palmiet | Palmiet | -34.09308333 | 19.05136111 | 2 |  |  |
| 136 | AC09C18 | Ratel | Ratel | -34.70725000 | 19.69733333 | 2 |  |  |
| 137 | AC09C19 | Ratel | Ratel | -34.67011111 | 19.69138889 | 4 |  |  |
| 138 | ES07A36 | Ratel | Ratel | -34.75330556 | 19.73047222 | 4 |  |  |
| 139 | AC09A32 |  | Uilkraals | -34.56222222 | 19.45513889 | 2 |  |  |
| 140 | AC09A33 | Uilkraals | Uilkraals | -34.57366667 | 19.47944444 | 4 |  | 1 |
| 141 | AC09A34 | Boesmans | Uilkraals | -34.59836111 | 19.59222222 | 4 |  |  |
| 142 | AC09A36 | Uilkraals | Uilkraals | -34.54366667 | 19.51519444 |  |  | 1 |
| 143 | AC09A37 | Uilkraals | Uilkraals | -34.52697222 | 19.53927778 | 6 |  |  |
| 144 | AC09A38 | Uilkraals | Uilkraals | -34.46708333 | 19.59711111 |  |  | 1 |
| 145 | AC09C11 | Uilkraals | Uilkraals | -34.51363889 | 19.61202778 | 2 |  |  |
| 146 | GZ42 | Slang | Uilkraals | -34.51338889 | 19.61216667 | 1 |  |  |
| Outgroups | *G*. ‘mollis’ | Leeu | Berg | -33.15591667 | 19.05058333 |  |  |  |
|  | *S*. ‘berg’ | Leeu | Berg | -33.15591667 | 19.05058333 |  |  |  |
|  | *P. asper* | Olifants | Gouritz | -33.62150000 | 21.94819444 |  |  |  |
|  | *P. tenuis* | Olifants | Gouritz | -33.62150000 | 21.94819444 |  |  |  |
