# Supplementary material for: Evolutionary Drivers of Diversification and Distribution of a Southern Temperate Stream Fish Assemblage: Testing the Role of Historical Isolation and Spatial Range Expansion
Source: PLoS One. 2013 Aug 9;8(8):e70953. doi: 10.1371/journal.pone.0070953 (PMC3739774; doi:10.1371/journal.pone.0070953)
Supplement: Appendix S2 — Distribution of Galaxias , Pseudobarbus and Sandelia lineages in the south-western Cape Floristic Region of South Africa. (DOCX) [file pone.0070953.s002.docx]

**SUPPORTING INFORMATION**

# **Evolutionary drivers of diversification and distribution of a southern temperate stream fish assemblage: testing the role of historical isolation and spatial range expansion**

Albert Chakona, Ernst R. Swartz and Gavin Gouws

**Appendix S2** **Distribution of *Galaxias*, *Pseudobarbus* and *Sandelia* lineages in the south-western Cape Floristic Region of South Africa.**

**
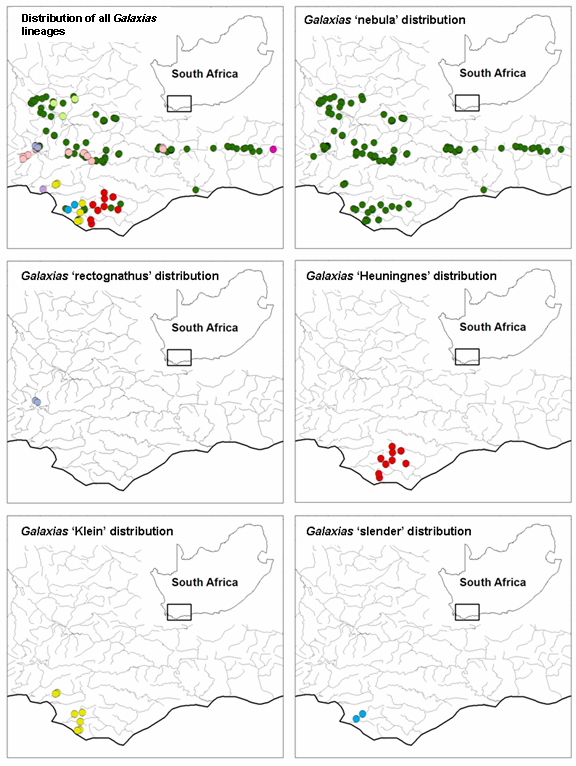
**

Figure S1 Distribution of *Galaxias* lineages in the south-western CFR. The colour codes are as denoted in Figure 4a.


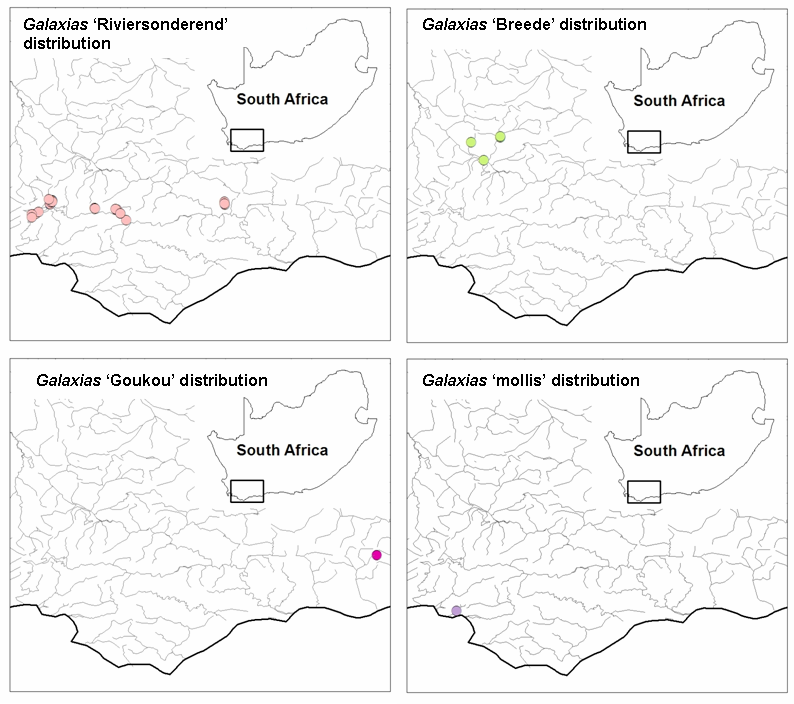


Figure S1 continued…


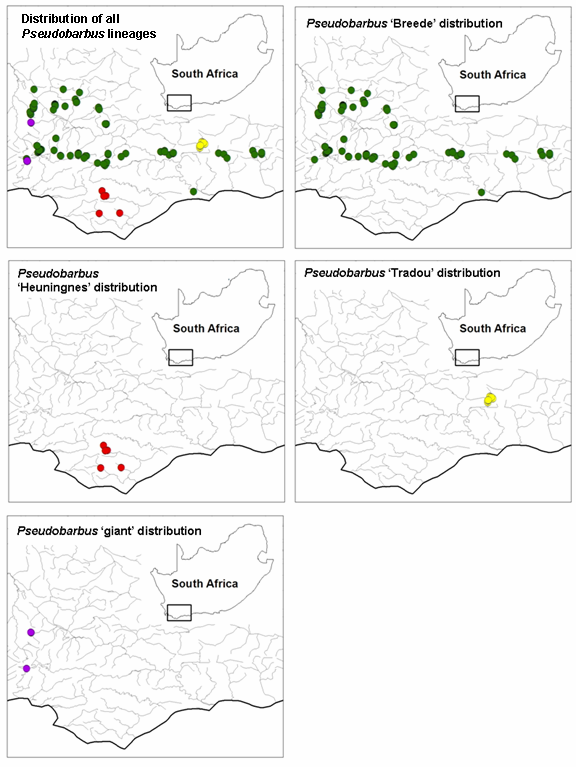


Figure S2 Distribution of *Pseudobarbus* lineages in the south-western CFR. The colour codes are as denoted in Figure 5a.


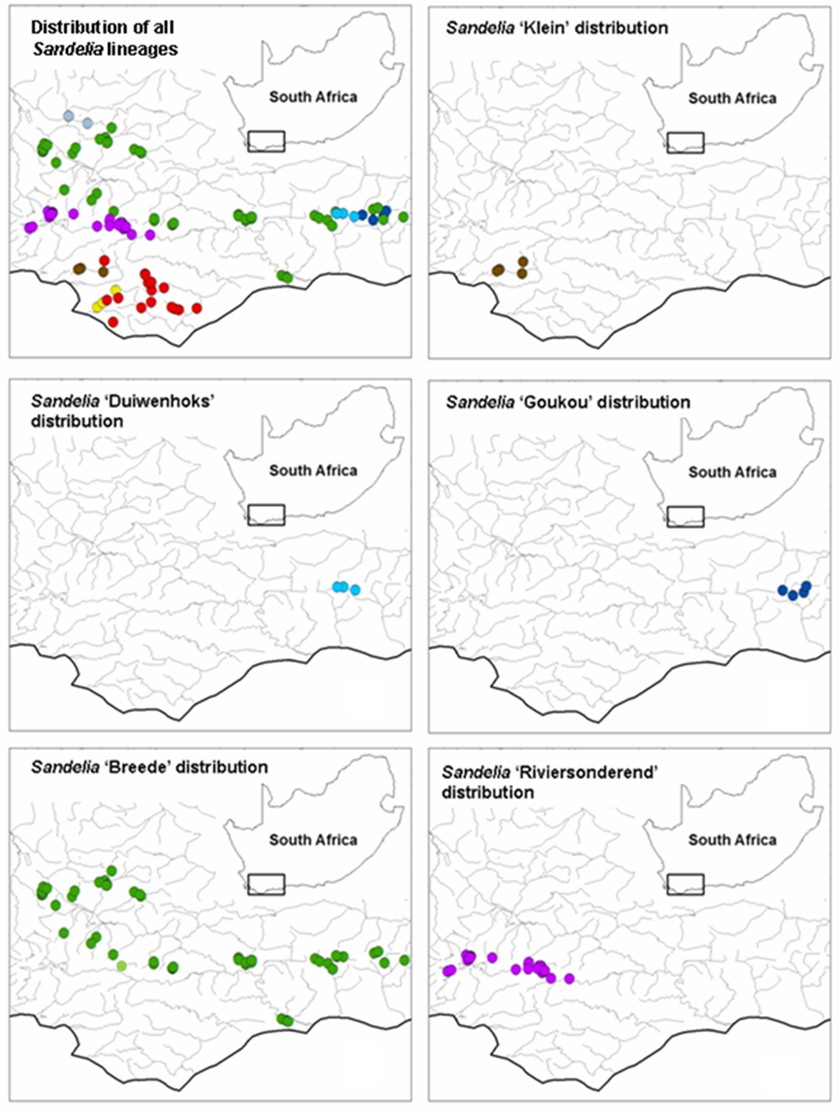


Figure S3 Distribution of *Sandelia* lineages in the south-western CFR. The colour codes correspond to the lineages as denoted in Figure 6a.


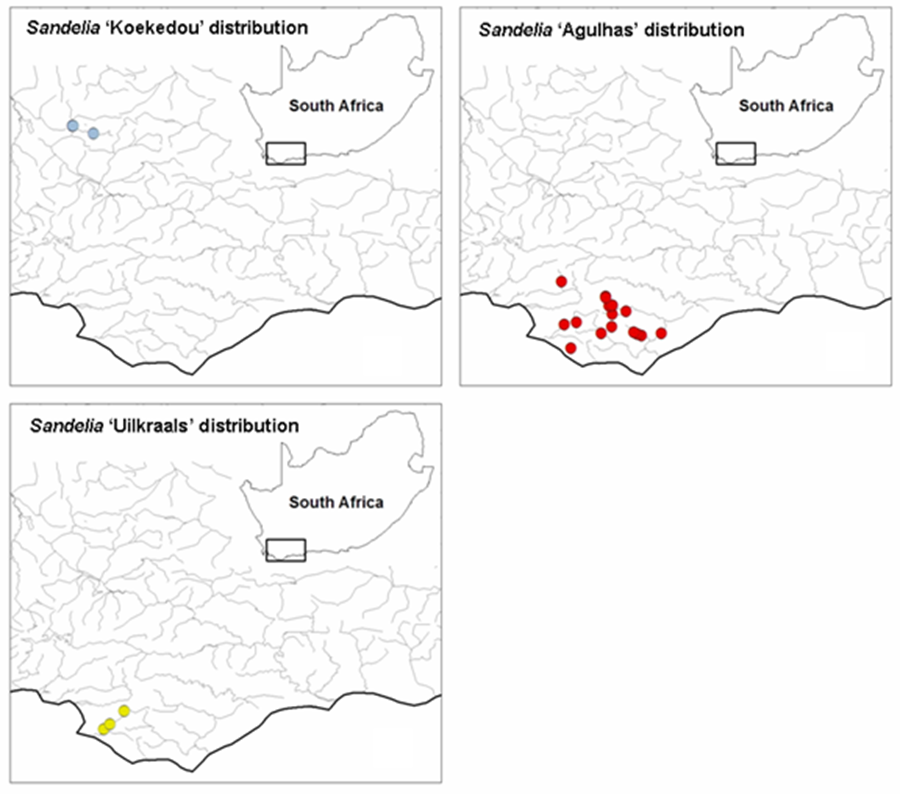


Figure S3 continued…
