# Supplementary material for: Evolutionary Drivers of Diversification and Distribution of a Southern Temperate Stream Fish Assemblage: Testing the Role of Historical Isolation and Spatial Range Expansion
Source: PLoS One. 2013 Aug 9;8(8):e70953. doi: 10.1371/journal.pone.0070953 (PMC3739774; doi:10.1371/journal.pone.0070953)
Supplement: Appendix S3 — Conceptual models for diversification and geographical distribution. Hypothesised processes that drove diversification and shaped the distribution of unique lineages within Galaxias, Pseudobarbus and Sandelia in the south-western Cape Floristic Region of South Africa. (DOCX) [file pone.0070953.s003.docx]

**SUPPORTING INFORMATION**

# **Evolutionary drivers of diversification and distribution of a southern temperate stream fish assemblage: testing the role of historical isolation and spatial range expansion**

Albert Chakona, Ernst R. Swartz and Gavin Gouws

**Appendix S3 Conceptual models for diversification and geographical distribution.** Hypothesised processes that drove diversification and shaped the distribution of unique lineages within *Galaxias*, *Pseudobarbus* and *Sandelia* in the south-western Cape Floristic Region of South Africa.


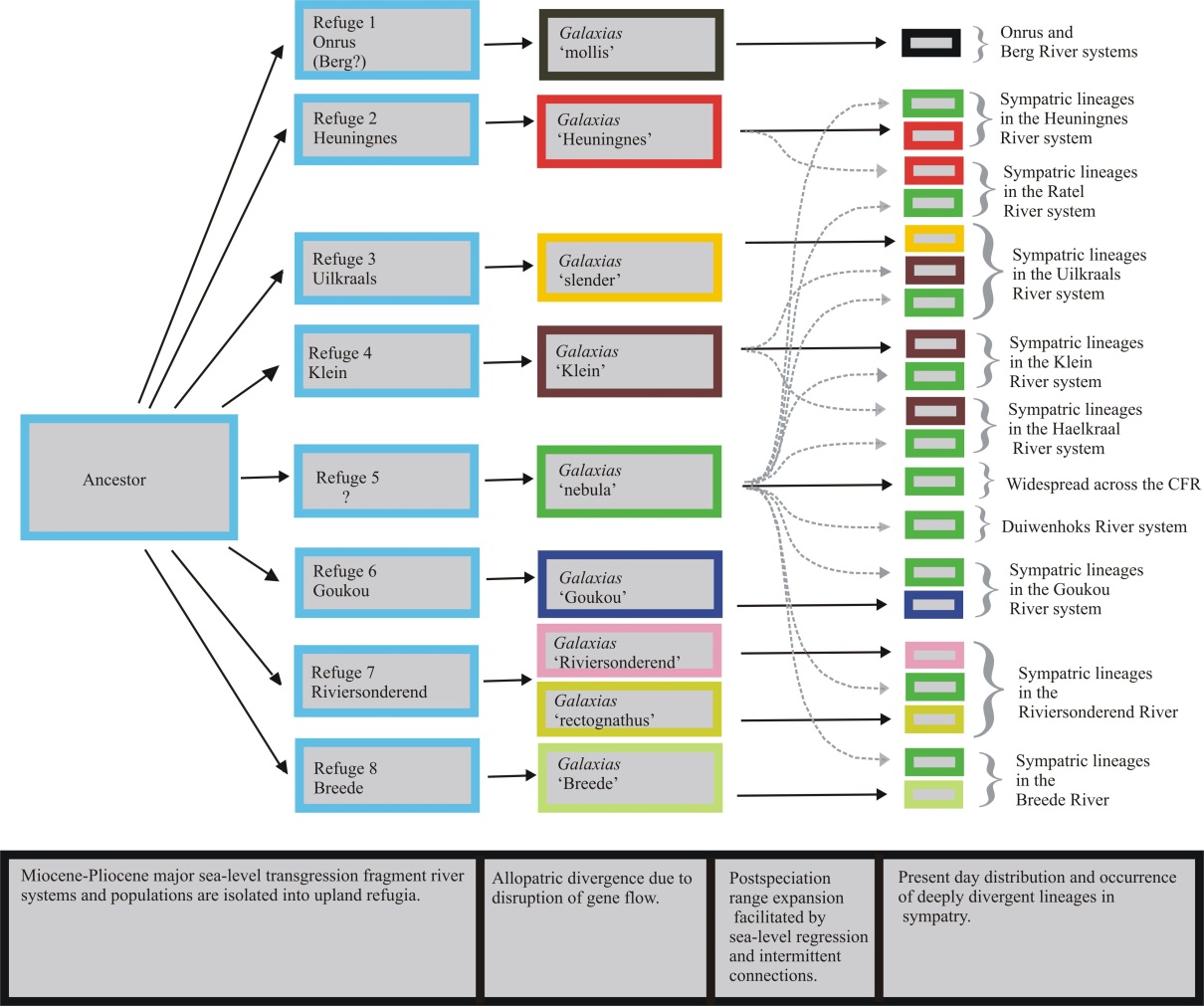


Figure S1 Conceptual speciation and distribution hypothesis for *Galaxias* in the south-western Cape Floristic Region.


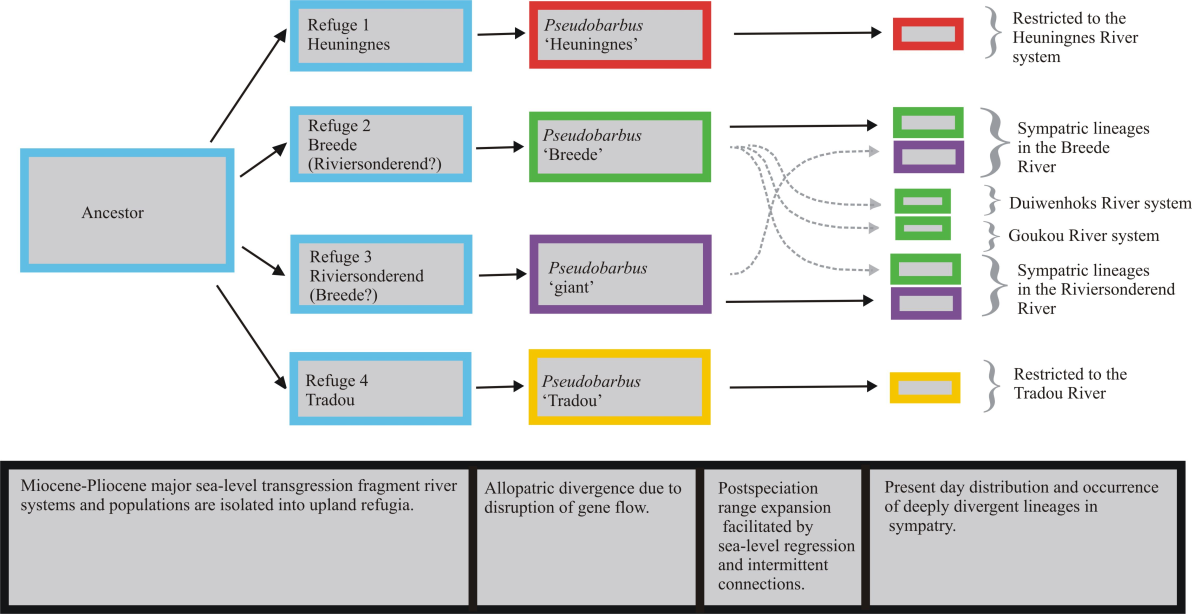


Figure S2 Conceptual speciation and distribution hypothesis for *Pseudobarbus* in the south-western Cape Floristic Region.


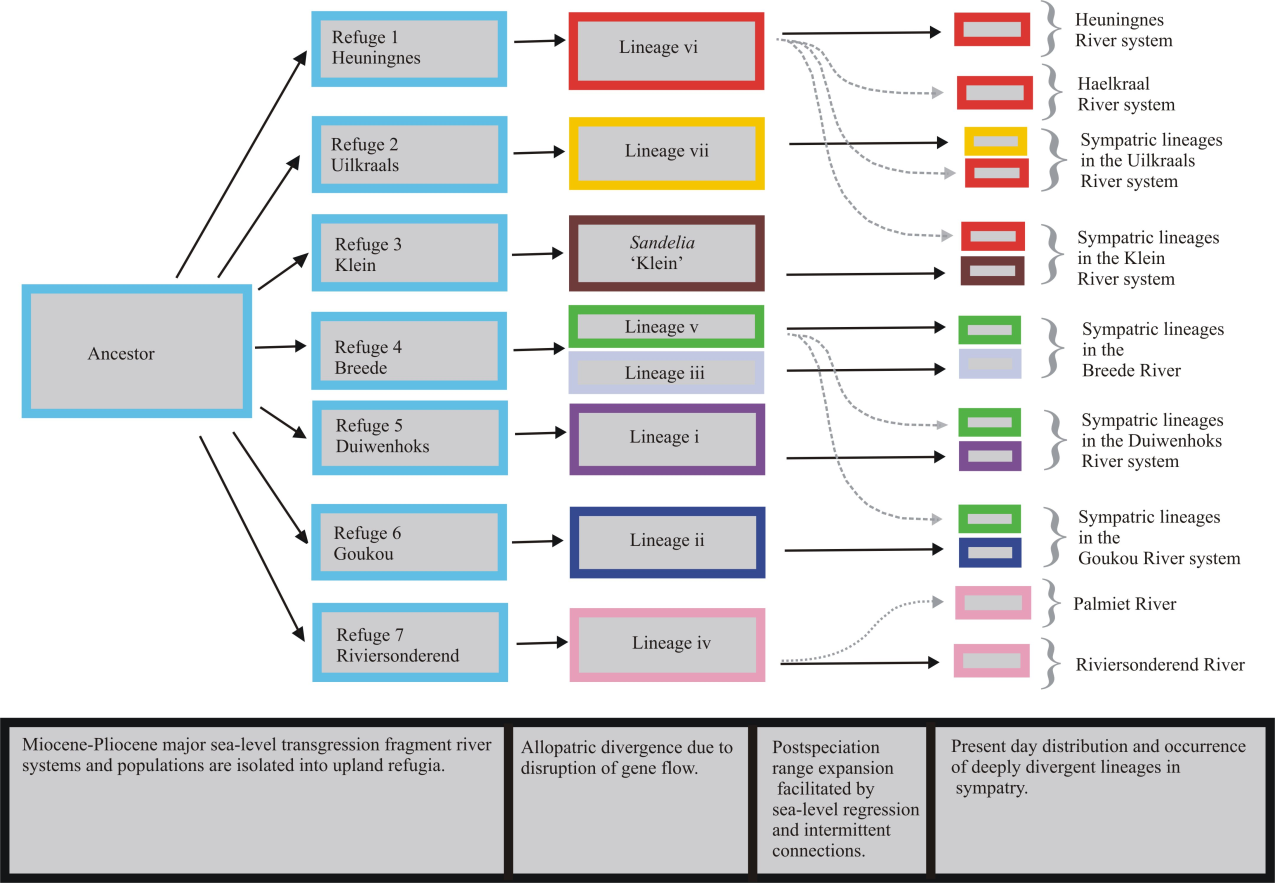


Figure S3 Conceptual speciation and distribution hypothesis for *Sandelia* in the south-western Cape Floristic Region.
